# e-VITA study protocol: EU-Japan virtual coach for smart aging

**DOI:** 10.3389/fpubh.2024.1256734

**Published:** 2024-03-12

**Authors:** Roberta Bevilacqua, Vera Stara, Giulio Amabili, Arianna Margaritini, Marco Benadduci, Federico Barbarossa, Elvira Maranesi, Anne-Sophie Rigaud, Sébastien Dacunha, Cecilia Palmier, Johanna Moller, Ryan Browne, Toshimi Ogawa, Rainer Wieching

**Affiliations:** ^1^Scientific Direction, IRCCS INRCA, Ancona, Italy; ^2^Université de Paris, Maladie d’Alzheimer, Paris, France; ^3^Services de Gériatrie 1 & 2, AP-HP, Hôpital Broca, Paris, France; ^4^Diocesan Caritas Assosiation of the Archdiocese of Cologne e.V., Cologne, Italy; ^5^Smart-Aging Research Center, Tohoku University, Sendai, Japan; ^6^Institute for New Media & Information Systems, University Siegen, Siegen, Germany

**Keywords:** older adults, virtual coach, protocol, intrinsic capacity, well-being, physical activity, frailty

## Abstract

**Aim:**

The aim of this study is to report a trial protocol for assessing the improvement of older adults’ well-being, promoting active and healthy aging, and reducing the risks of social exclusion, using a virtual coach.

**Background:**

Increased longevity brings with it reduced autonomy and independence, and it is therefore necessary to act with preventive measures that can promote active and healthy aging. With the development of technology, new tools have appeared, including virtual coaches, which can enable people to lead a healthy lifestyle by identifying individual needs and goals and providing personalized recommendations and advice. However, it is important that these coaches take into consideration the inter-individual and cross-cultural differences of each person.

**Design:**

A randomized controlled trial is proposed.

**Methods:**

This study will recruit 240 healthy subjects aged 65 years and older. Participants will be assigned to an experimental group that will receive the e-VITA system or to the control group that will receive an information booklet only. The primary outcome measure is the person's quality of life (QoL). Data will be collected at baseline, 3 months after the trial, and at the end of the trial, after 6 months.

**Discussion:**

This study will evaluate the effectiveness of the e-VITA system, consisting of a virtual coach, several sensors for monitoring, a smartphone for use at home, and a booklet, in improving the older person's quality of life. The increased perceived well-being will also be linked to improvements in other areas of the person's life, psychological and cognitive status, the area of sociality, nutrition, and eHealth literacy.

## Introduction

Aging brings with it some chronic conditions, along with the reduction of some functions, (1–3) which can lead to a loss of autonomy and frailty. The care of older adults with a loss of autonomy is therefore becoming a major social, medical, and economic issue due to the insufficient number of family or professional carers, enabling older adults to live at home as long as possible and delay entry to institutionalization (4). However, aging is not the same for all individuals. It is dependent on many factors such as genetic factors that cannot be modified and environmental factors including lifestyle (5). It is on the latter that preventive actions can be carried out throughout life (1). In addition to preventive action, the development of new care solutions is important (6), to reduce or minimize the consequences of these age-related diseases, support the health system, and promote active and healthy aging.

## Background

Europeans are living longer and longer. According to INED (7) the life expectancy of a European woman is 83.7 years and 78.3 years for a man in 2018. Japan is the country with the highest life expectancy. Women live on average 88.1 years and men live on average 81.9 years (7). However, the increase in life expectancy is not necessarily accompanied by an increase in healthy life expectancy. In Europe, a woman can expect to live in good health for an average of 64.5 years and a man can expect to live in good health for an average of 63.4 years (8).

As people age, some of their cognitive functions deteriorate (9), and they experience psychological and physical (10–12) difficulties. These changes generate an increase in the workload required by both family and health professionals (13, 14). For these reasons, it is necessary to act with a view to prevention, starting with the concept of active aging. Active and healthy aging refers to the maintenance and development of functional abilities that enable older adults to live well (World Health Organization, n.d) in terms of physical, mental, and social health while actively participating in society.

With the emergence of the concept of healthy and active aging and the identification of factors that contribute to the development of age-related diseases and loss of autonomy, health organizations have tried to improve their strategies to prevent these risk factors and diseases (15). Different structures, organizations, or professionals offer prevention services such as physical, cognitive, emotional, or social activities. However, access to private professionals for prevention targeted to individual needs and preferences is expensive, whereas the offer of public organizations is only slightly customizable and may not be accessible to all seniors. This inequality of access is reinforced by the current health context linked to the COVID-19 crisis, which has led to the cessation or postponement of workshops or face-to-face activities in sports and intergenerational or cultural clubs. A number of videoconference activities have been developed over the past years. However, this remote format does not facilitate the personalization of interventions. With the development of technology, new tools have appeared, including virtual coaches, which seem to be of interest in supporting the behavior of individuals. A virtual coach is defined by Siewiorek et al. (16) as a personalized system that continuously monitors the activities and environment of its users. Virtual coaches detect situations where an intervention would be desirable and proposed to the user. To this end, coaches take the form of activity sensors combined with a coaching application located on the Internet, a smartphone, a sensor (17), or a social assistance robot (SAR) (18). Technology plays an important role in the aging process, impacting both individuals and society as a whole. On a personal level, wearable gadgets and health applications keep track of one’s well-being, and telemedicine offerings enhance the availability of healthcare services. At the societal level, health efficiency, data-driven policies, and the development of social platforms connecting users have a positive influence. However, challenges such as equitable digitization and accessibility require attention to ensure a beneficial and inclusive impact, especially considering the skill level of the users of older than 65 years with technology, not only from a user skills perspective but also from a tool acceptance perspective (19, 20). In this regard, several studies have been conducted that quantitatively and qualitatively assess access to the Internet of Things (IoT) among the older adult population (21, 22).

Through a personalized approach, the virtual coaching system can enable people to live a healthy lifestyle, identifying personal needs and goals and providing appropriate risk predictions and individualized recommendations (23, 24). These devices cover a variety of domains and audiences (25). There are a multitude of virtual coaches and apps for the promotion of nutrition (26), physical activity (27), mood, sleep (28), and even more clinical applications, such as rehabilitation (29) or monitoring of cardiovascular pathologies (30).

There are virtual coaches for adults, children with autistic disorders, and older adults. These tools have attracted interest from healthcare organizations and consumers for promoting health, wellness, physical activity, and lifestyle improvement (31). The use of these virtual coaches and apps has great potential to help older adults improve their quality of life by addressing age-related issues and the physical and social implications of aging (32). Moreover, embracing these proactive measures with the assistance of a virtual coach empowers older adults to maintain independence and comfortable living in their homes for an extended period (33). This reduces the need for constant monitoring by health professionals, limits the costs that would otherwise be incurred (32), and helps mitigate the wider impact of demographic aging on healthcare (4).

However, the technologies developed for healthy and active aging have some limitations. Indeed, they are mainly used for short period of time and are poorly integrated into the daily lives of older adults, thus limiting their benefits (6). Moreover, interactions with these technologies are not ideal since they do not lead to realistic and satisfying social interactions due to technologies that are not yet advanced enough (34). It is therefore necessary to design and evaluate new products that consider the needs and preferences of seniors and their relatives for a sustainable and optimal use of these devices (35). The aim is to enable seniors to live independently in their own homes for as long as possible, preventing social isolation and encouraging active participation in social and group activities.

In this context, the creation of an intercultural and customizable virtual coach may represent an effective solution. Through cooperation between European (Italy, France, and Germany) and Japanese partners, the e-VITA project proposes different technological tools that are adapted to older adults and their daily lives, investigating the effect of cultural aspects in accepting technology and how this affects the outcome. In tackling the task of crafting these technological tools, the e-VITA project takes on a comprehensive approach. This involves seeking input from all stakeholders at both the personal and societal levels. By engaging older adults, their informal and professional health caregivers, community members, and policymakers, the project aims to foster collaboration. The insights gained from these consultations are then mapped and utilized to comprehend the contextual use and identify potential users of the system (36, 37). The inclusion of different types of beneficiaries in all the countries could support local governments to adopt and adapt cross-national policies for AHA that fulfill the real needs of older adults, respecting different cultural perspectives. Therefore, the functionalities and services available and proposed are in response to the needs of older adults and concern social relations, physical activities, skills, autonomy, stimulation, communication, and safety.

## The study

### Study setting

The study will be conducted at the Center for Cognitive Disorders and Dementia (CDCD) of the Neurology Unit of the Istituto Nazionale Ricovero e Cura per Anziani IRCCS INRCA, Ancona, Italy. In Germany, the study will be carried out by the Diözesan-Caritasverband für das Erzbistum Köln e.V. (Diocesan Caritas Association for the Archdiocese of Cologne e.V.). In France, the study will be conducted by Hôpital Broca, Paris. In Japan, three institutes will carry out the study, namely, Tohoku University –Smart Ageing Research Center, J.F. Oberlin University, and Misawa Homes Institute of Research and Development Co. Ltd.

### Aim

The research is structured as a randomized controlled trial with the objective of enhancing well-being in older adults, promoting active and healthy aging, contributing to independent living, and mitigating the risks of social exclusion through the utilization of a virtual coach. Participants will be divided into two groups: the experimental group (EG), receiving a virtual coach, a smartphone, and a booklet, and the control group (CG), receiving only a booklet.

Assessments will be conducted at the baseline (T0), midway through the intervention (after 3 months—T1), and at the conclusion of the intervention (after 6 months—T2). The primary aim of the study is to gauge the enhancement of participants’ quality of life (QoL) using EQ 5D 5 L. Additionally, the research will assess the usability, user experience, acceptability, and fulfillment of needs after using the e-VITA system. It will also explore potential changes in various health-related aspects, such as nutrition, loneliness, and health literacy.

### Objectives

The general objective is to improve the well-being of older adults, promote active and healthy aging, contribute to independent living, and reduce risks of social exclusion of 240 healthy older adults recruited from Europe (France, Germany, and Italy) and Japan by making use of a digital tool for personalized virtual coaching support.

### Design/methodology

#### Recruitment

Patients are selected by the Neurology Operating Unit and Rehabilitation Medicine Operating Unit of IRCCS INRCA in the Ancona branch. After a reflection period of 2 weeks followed by reading the information letter and the consent form received by email, the participants who confirmed their wish to participate in the research by writing or orally will be invited by the investigator to consult a doctor during 3 months, preceding the start of the research. The doctor, volunteering to carry out the inclusion visits, will be the first to give the participants an information letter and two consent forms. Once the consent forms have undergone thorough proofreading and received signatures from both the participant and the doctor, the doctor will assess the participant’s eligibility for the research using an anamnesis Clinical Frailty Scale (CFS) (38), Montreal Cognitive Assessment (MOCA) (39), Geriatric Depression Scale (GDS) (40), and Short Physical Performance Battery (SPPB) (41). This comprehensive medical examination, conducted before the commencement of the experiment, will determine whether participants meet the inclusion criteria. Those who have signed the consent form but do not meet the inclusion criteria as confirmed by the doctor will be excluded from the research. The trial is scheduled to commence in May 2023 and is anticipated to conclude in December 2023.

#### Participants

Each European country will enroll 40 subjects.

The inclusion criteria are:

- Age ≥ 65 years;
- Ability to provide informed consent;
- Able to stand and walk unaided;
- No acute or untreated medical problems;
- MoCA ≥22;
- GDS < 9;
- SPPB ≥7;
- Clinical Frailty Scale score between 2 and 4;

For patients with MoCA scores between 22 and 25, an informal caregiver is required to be present during the explanation of the project and the administration of the assessment scales.

The exclusion criteria are:

- Failure to meet the inclusion criteria;
- Use of active implant or non-implant medical devices;
- Allergy to nickel;
- Simultaneous participation in other studies;
- Absence of written informed consent;
- Occurrence of a myocardial infarction or stroke within the past 6 months;
- Presence of painful arthritis, spinal stenosis, amputation, painful foot lesions, or neuropathy that significantly limits balance and mobility;
- Uncontrolled hypertension;
- Presence of a pacemaker or implantable cardioverter-defibrillator;
- Advanced Parkinson’s disease or other neuromuscular disorders;
- Diagnosis of metastatic cancer or undergoing immunosuppressive therapy;
- Significant visual or hearing impairment.

#### Sample size determination

In the study by Summers et al. (42), the EuroQol 5 dimensions (EQ 5D) was evaluated in two groups of older adults with physical pre-frailty conditions. EQ 5D, a test widely used to measure health status-related quality of life, was used to calculate the sample size. Assuming an overall sample size of 220 subjects (110 cases and 110 controls), 2 repeated assessments (baseline and follow-up), a significance level of 0.05, power of 90%, a correlation among repeated measures of 0.5, and a non-sphericity correction ε of 1 in an ANOVA model within-between interactions, the achieved effect size for this study is 11% (corresponding to a small effect size in a coherent way with the literature). Even factoring in a dropout rate of 10%, the total number needed would be 240 subjects, evenly divided into 120 cases and 120 controls. The hypothesis is that this sample size is more than adequate to detect variations in secondary outcomes. For these secondary outcomes, a treatment effect size is assumed to be similar to or even greater than the one identified for the primary outcome.

#### Intervention

**Figure 1:**
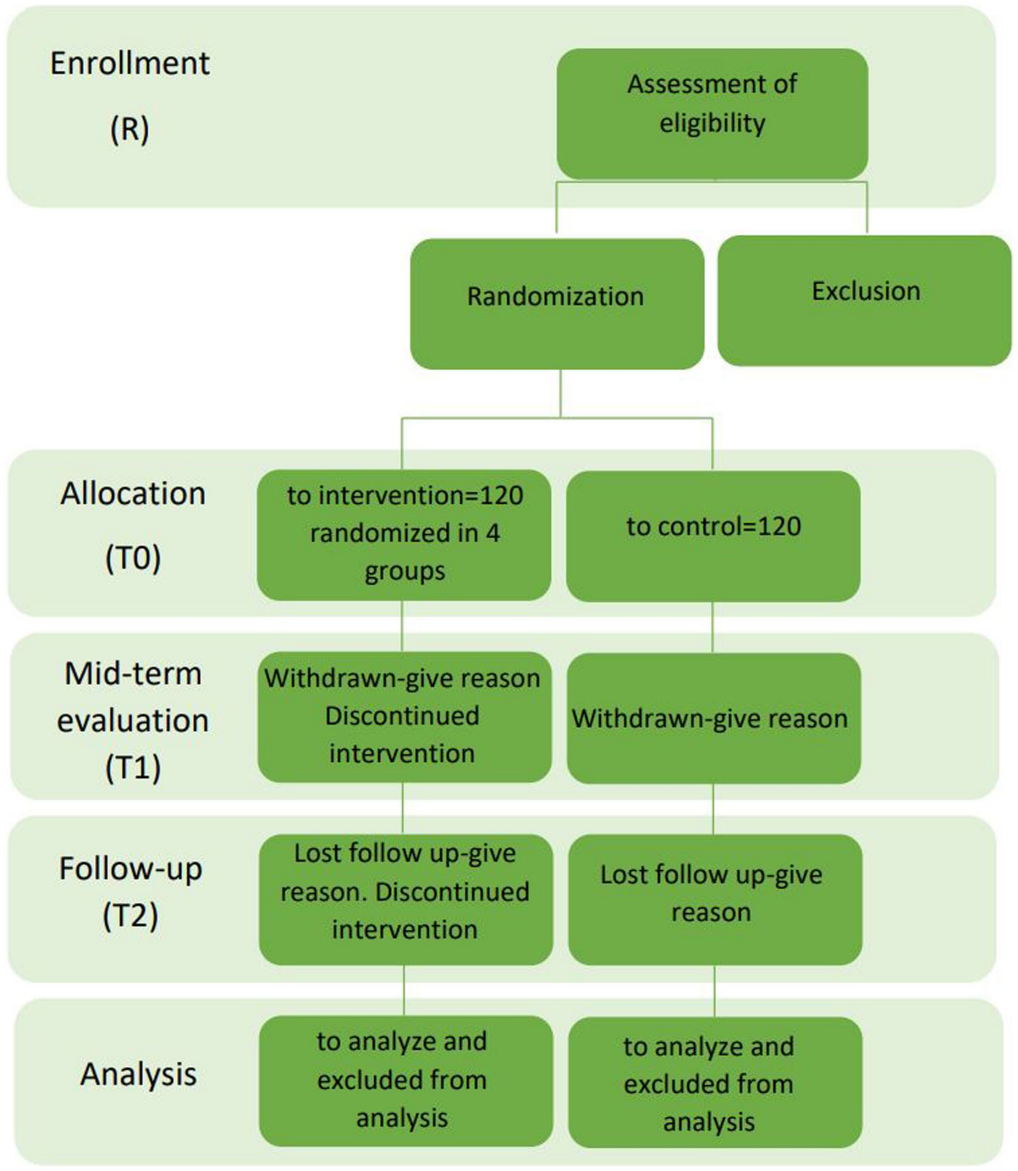
Flowchart of the patient selection.

The interventions are oriented toward the model of intrinsic capacity (43), and an improvement of the general well-being is to be achieved through the promotion of the different areas. For this study, 240 healthy older adults will be enrolled. Figure 1 illustrates the flowchart, detailing the process of patient selection.

In total, 120 subjects will be enrolled in the experimental group and will receive the e-VITA platform composed of a virtual coach, several sensors for monitoring, and a smartphone to use at home.

The control group will receive only the booklet with nutritional, cognitive, and physical suggestions.

### Equipment

An innovative Information and Communication Technology (ICT)-based virtual coaching system has been created to identify subtle changes in the physical, cognitive, psychological, and social domains of older adults. The e-VITA virtual coach is designed to offer personalized recommendations and interventions, fostering sustainable well-being within a smart living environment at home. All the devices will be supplied by the researchers.

The different components of the system are:

- Coaches, consisting of social robots, that will interact with the users and are guided by apps;
- Sensors (both wearable and domestic) to detect physiological parameters, physical activities, and behavior of the users; these sensors are the Huawei smart band (wearable), the NeU device (wearable), and the DeltaDore system (domestic).
- Smartphones (the chatbot to provide insights, suggestion, and stimulation about healthy nutrition and physical exercise; the social platform to encourage users to share their interests).

These components (coaches, sensors, chatbot, and social platform) together with the main software named Digital Enabler (DE) constitute the Virtual Coach. The DE takes into account different types of data (both from the literature and different devices described above together with the user’s personal data), to make choices in order to customize the user experience. Choices are made both on the basis of the data received and the use case considered. The DE operates directly on the sensors, robots, and chatbots.

Categorizing sensing technologies and coaching devices based on their inputs is integral to establishing a cohesive and integrated sensor network. This network possesses the ability to recognize user behaviors, physiological states, and emotions, ultimately pinpointing a coaching device that resonates with the user’s preferences and acceptance.

Three categories of sensors could be distinguished: (1) those which are worn by the user and aim to sense physiological and actimetric parameters (user-related devices); (2) those which measure physical quantities useful for assessing the level of comfort and quality of the indoor environment and localization of the user (environmental devices); (3) those installed in the home to monitor user behavior and activities (home-based devices). For the e-VITA system, all these types of sensors are combined to make inferences about simple situations of the users (posture, activity level) in their environment and for localization in the home and the users’ physiological states. Contextual information is exploited by the interactive voice-based coaching system, the virtual coach. Each domain requires the integration of specific sensors to acquire heterogeneous data, and a coaching device is selected based on the user’s information, preferences, and technical characteristics and functionalities performed. This task is performed by the Use Cases Configurator, a tool that aims at identifying the optimal configuration of the sensor network and coaching devices to be used based on the user’s needs and requirements. The coaching devices can take four forms, namely, Gatebox, Nao, Google Nest Hub, and Celeste (or DarumaTO for Japan). The specific mobile applications for the usage scenarios are available on the Android smartphone, which is provided to the user as a support device.

### Technological description of coaches

The coaching devices used in the study are Nao Robot, Gatebox, and Celeste, substituted by DarumaTO for the Japanese centers (Table 1).

- Nao is a humanoid Robot exhibiting a friendly appearance with eyes and movements reminiscent of a human being. Nao is equipped with an array of sensors, cameras, and microphones, enabling it to perceive and interact with its surrounding environment and human beings. It is powered by advanced artificial intelligence software, enabling capabilities, such as facial recognition, natural language understanding, and learning from user interactions (44–50).
- Gatebox is an intelligent home assistance device developed that appears as a holographic projector embedded in a cylindrical container, housing a virtual editable characteristic. This characteristic is a virtual assistant equipped with artificial intelligence, designed to interact with users akin to a personal assistant. From a technical standpoint, Gatebox utilizes holographic projection technologies to display coach characteristics within a three-dimensional environment. It is equipped with microphones and speakers to recognize and reproduce sound, allowing users to communicate with the virtual assistant through voice recognition and audio responses. Gatebox can perform a variety of tasks, such as providing information related to physical activity recommendations and giving dietary advice. Moreover, it is designed to provide a more intimate and personal interactive experience compared with other virtual assistants, incorporating elements of relationship and affection in its behavior (51–55).
- CelesTE (56–58) and DarumaTO (59–61) are social robots that have religion as a principal theme. CelesTE resembles an angelic statue atop a column inspired by sacred Christianity art and neoclassical architecture. This social robot incorporates the golden ratio to transcend its robotic nature and evoke a sense of holiness. Primarily designed as a “guardian angel” for older adults, CelesTE serves as a prayer companion and repository of religious teaching, including the Bible. AI enables short conversations on sensitive topics and can print selected content. DarumaTO is inspired by the traditional Buddhist and Shinto doll called Daruma. It can communicate through visual tracking and voice and facial expression. Its functionalities are similar to those of CelesTE, but in the case of design, it results in a device that has a familiar appearance to a Buddhist or Shinto older adults.

**Table 1:** Coaches.

Name	Type	Description	Main functionalities
NAO robot	Coaching device/Robot	Softbank NAO 5 and NAO 6 humanoid interactive mobile robot.	Robot platform that allows multimodal natural language interaction and robot autonomous movement.
Google Nest Hub (2e Generation)	Coaching device/Virtual assistant	Connected speaker enriched with a 7-chip touch screen	Screen whose brightness adapts to the room’s atmosphere. It has a loudspeaker and 3 microphones, making interaction possible.
Gatebox	Coaching device/Hologram	Hologram like device that projects characters with which the user can interact.	Internal sensors such as a camera and a microphone allow the user to converse with the projected character. It connects to the Internet via a wireless LAN. With infrared rays and Bluetooth, it can also be connected to household appliances and other devices.
CelesTE	Coaching device/Robot	Prayer companion designed for Christian Catholic users.	The intended main function of CelesTE is to be a “guardian angel,” especially thought for older people. It can be a prayer companion, and contains a vast number of teachings, including the whole Bible. Its AI is capable of keeping a short conversation, in which the user may ask and receive an answer about a sensitive topic (such as happiness, death, faith, etc.). It can also printout a selection of contents.

Such devices are in charge of interacting with the user, exploiting the dialog features provided by the platform.

The randomization technique, performed by the statistician, relies on a singular sequence of random assignments. A computer-generated list of random numbers is employed, and each subject is assigned a number based on their inclusion order in the study. Following this method, subjects are randomly allocated to the utilization of various coaching devices, maintaining an allocation ratio of five subjects for each branch.

### Technological description of sensors

The sensors are wearable and environmental, and they are needed to monitor physiological and environmental parameters. Table 2 presents the sensors available for the study.

**Table 2:** Sensors.

Name / Type	Description	Main functionalities	
DELTA DORE Tydom platform	IoT Device/Sensor	Set of sensors and measuring devices and IOT platform. The sensors can be used with a gateway connected to any dedicated platform.	Set of smart living sensors (motion sensors, door sensors, metering devices) and IOT platform.
Samsung Galaxy S20+ Smartphone	IoT Device	Android smartphone	To access all the apps and services
Smart band Huawei	IoT Device/Sensor	Wearable smart band that tracks physiological parameters.	Parameters monitored: activity level, step, calories, sleep duration, sleep quality, etc.
NEU XB-01	IoT Device/Sensor	Ultra-compact device with a butterfly-style design that bends in the middle to conform easily to any individual’s forehead. Data is transferred via Bluetooth in real-time to any smartphone, making it possible to measure brain activity.	Brain activity is measured using NIRS technology and the brain’s rate of blood flow change is measured using weak near-infrared light.

On the environmental sensors, Delta Dore motion detectors can detect the presence of a moving person in a room, which provides information on the occupancy of rooms and the movement of occupants from one room to another. This information can be of interest as a complement to the wearable sensors, especially when they are forgotten or under load. In this case, it is possible to use environmental sensors, especially motion sensors, to infer user activity. The sensor network, especially the infrared-based presence detection sensors (PIR sensors), should be strategically installed to effectively monitor the user, particularly in the designated areas of interest within the smart home environment. A predetermined selection ensures coverage of specific rooms. The primary objective of this system is to initially recognize the resident’s movements and potentially infer activities within a particular room of the apartment. The Use Case Configurator aggregates all the information detected by the environmental sensors, serving as input to feed the algorithms. Those sensors are:

- The Wireless motion detector-Delta Dore DMB TYXAL. It is a motion sensor and not a presence detector. It has a range of 12 m with a 90⁰ opening angle. It generally covers one room, and detection is limited to opaque walls. Its autonomy is of the order of 10 years. The detector is based on passive infrared technology. The motion detection function consists of a pyroelectric sensor, the associated electronics, which process the signal and control the sensor power supply in an optimized way, and finally a lens.
- The Opening magnetic sensor–Delta Dore DO TYXAL+. DO TYXAL+ is an opening sensor developed to detect intrusion in dwelling when doors or windows are opened. It consists of two parts, one of which is attached to the door or window jamb, and this is the active element. The other part is a mechanical part that contains a magnet and is attached to the moving part of the door or window. These parts are attached to the doors with a double-sided tape. The sensor consists of a reed switch on the active element which is closed when the magnetic part is closed and opens when the magnet moves away from it, i.e., when the door is open. The technology is simple, robust, and energy-efficient.
- The Tydom Home. Sensors are battery-powered and communicate detection and maintenance information to a central unit. The Tydom Home IP gateway will be used to collect data from the various products and transfer them to the e-VITA configurator.

### Description of the end-users’ platform

The end-user’s platform is composed of a privacy dashboard, smartphone app, Chatbot, social platform, and use case configurator. Through those applications, the end-users will be capable of managing his/her data and receiving information on health activities and social events. However, the virtual coaches represent the main interface of the system through vocal communication with the end-users.

**Figure 2:**
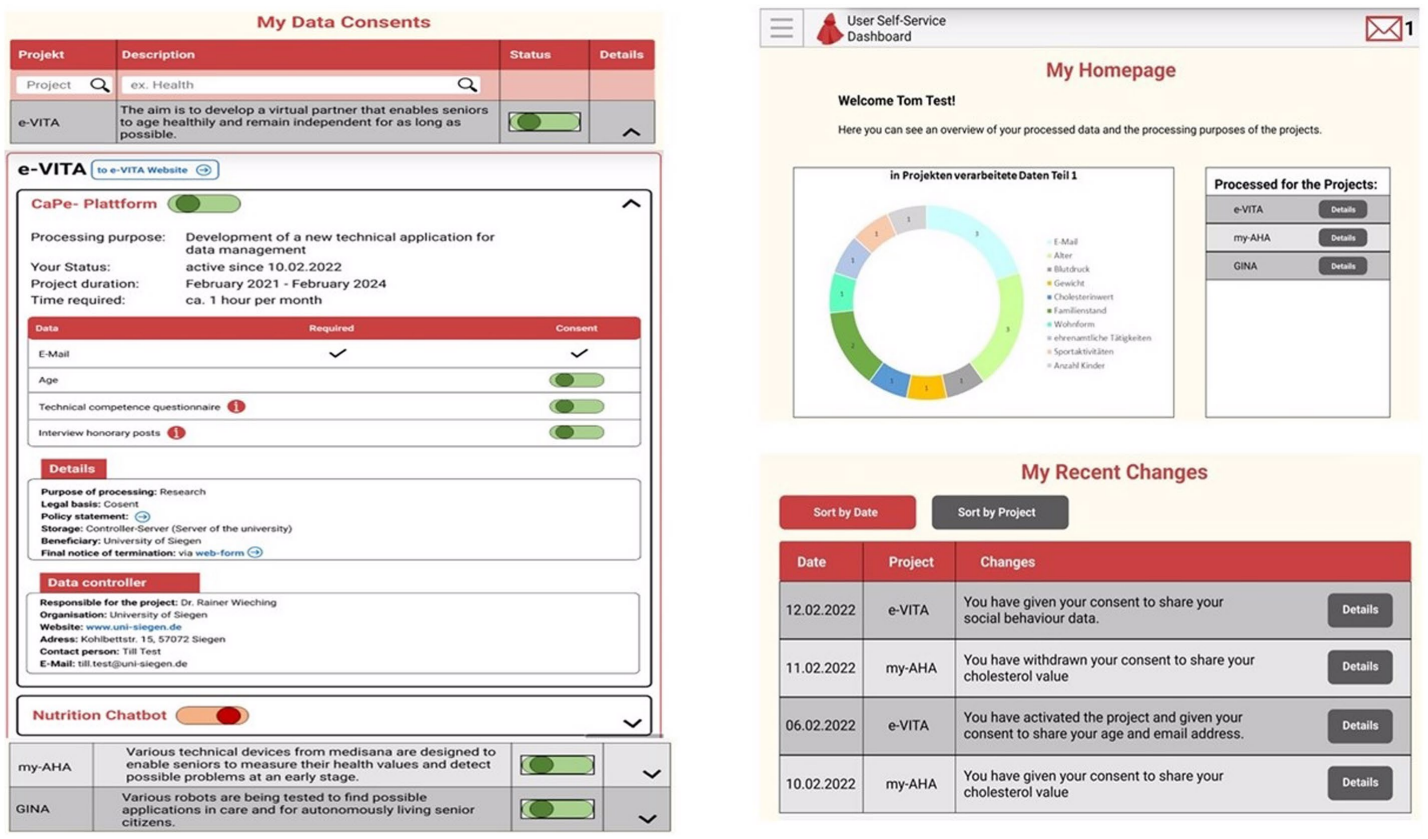
Screenshots of the privacy dashboard, where user can manage permissions and find information about data sharing.

**Figure 3:**
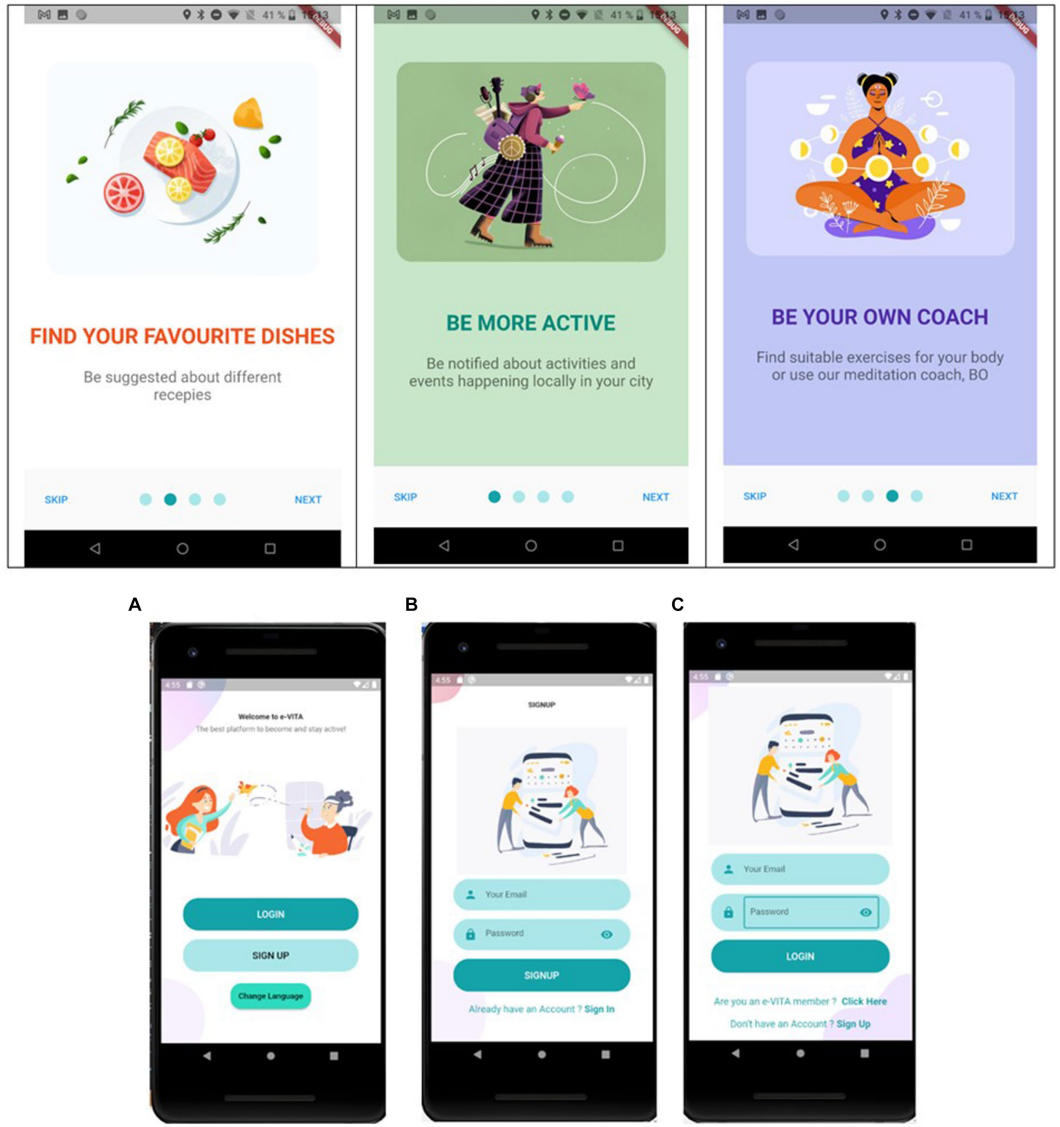
e-VITA dashboard access page that is shown when opening the app **(A)** landing page, **(B)** login page, and **(C)** sign up page.

**Figure 4:**
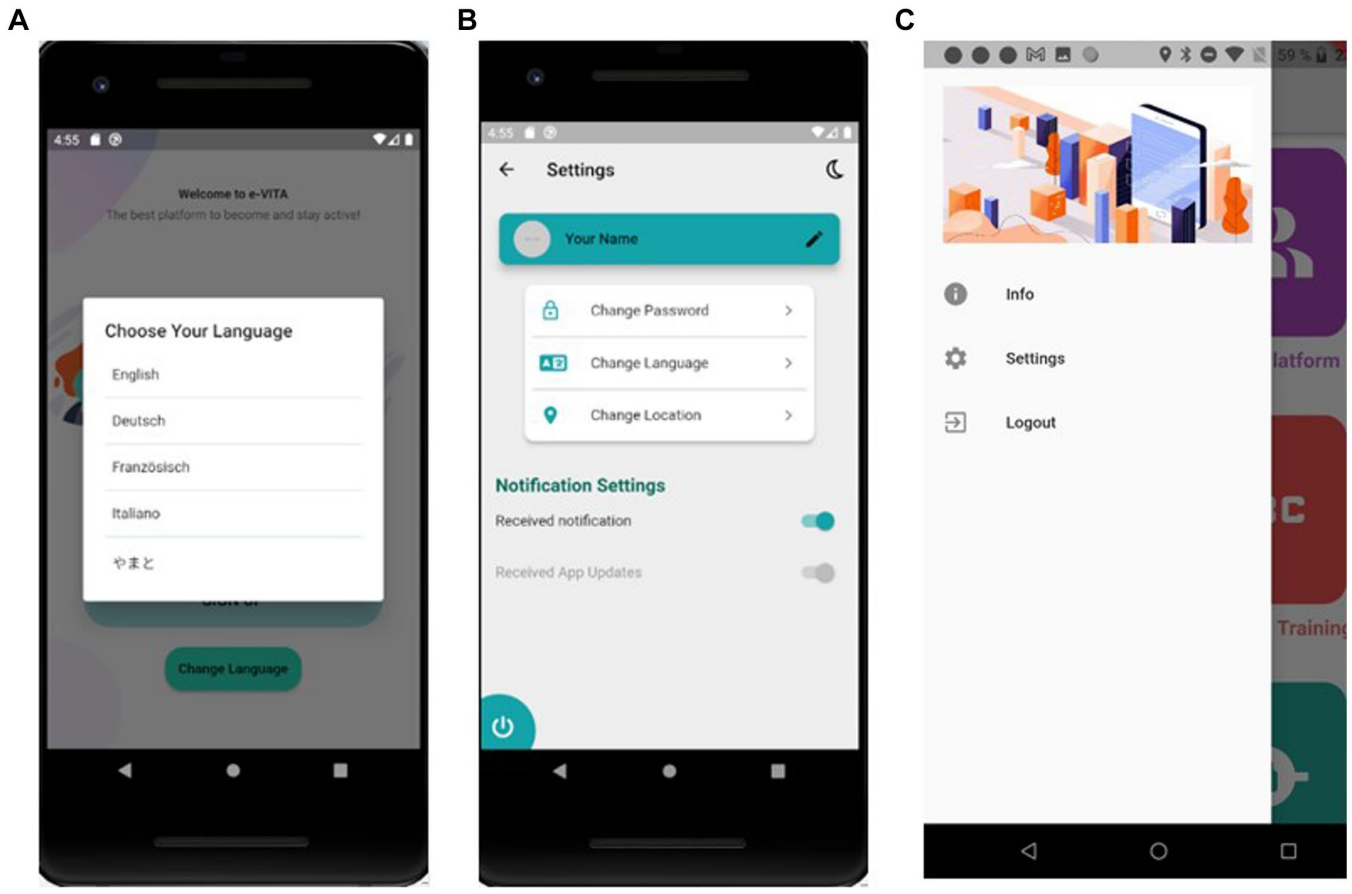
e-VITA dashboard settings control—**(A)** language settings, **(B)** settings page, and **(C)** dashboard lateral menu to access settings.

**Figure 5:**
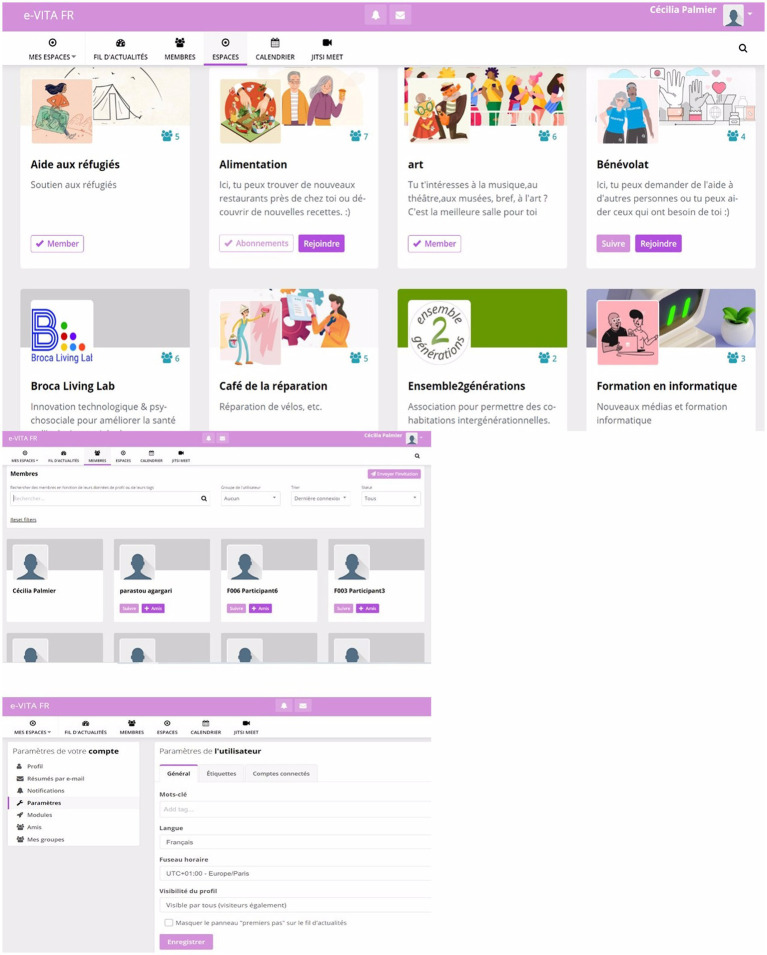
e-VITA dashboard homepage where all the apps and services are shown and accessible.

- *Privacy Dashboard*: CaPe offers a privacy dashboard through the Cape Suite, compatible with any mobile device. This system is designed to adhere to legal requirements outlined in the GDPR. It incorporates consent-based data management, technical mechanisms for verifying compliance with data handling prescriptions, the right to obtain a copy of personal data, the right to be forgotten, and transparency tools on data usage. The consent record maintained by CaPe details who consented, when the consent was given, what was consented, how the consent was granted, and any instances of withdrawn consent. CaPe supports two types of consenting: (1) consenting to process within a service for a specific purpose and (2) consenting to share data from a service (source) to be processed in another service (sink) for a specific purpose. The Cape Suite features two frontend dashboards, catering to end-users (data subjects) and service providers (data controllers), respectively. The User Self-Service Dashboard serves as a centralized point for end-users to overview, verify, and modify data usage settings, understand the purpose of data processing, view event logs, and adjust linked services and consents previously granted. On the other hand, the Data Controller Dashboard is the entry point for service providers, allowing them to manage semantic descriptions and registrations of their provided services and view and manage service linking and consent status given by all users of their registered services. Some screenshots of the dashboard are shown in Figure 2.
- *Smartphone App and Chatbot*: The e-VITA project provides a smartphone app for end-users that functions as the control center. From there, users can access all relevant apps and interventions. Furthermore, apps that offer control over system settings, such as the privacy dashboard, can be reached via the control center. Specific e-VITA apps (e.g., social platform), but also external apps, are represented on the dashboard. The app requires a user profile to be accessed. After opening the app, users will observe an introduction to app which can be skipped to the landing page (see Figure 3A). From there, e-VITA users can create a profile using their email address and a secure password (see Figure 3B). Additional information is not needed for the sign-up process. The email and password will be used to log in and access the individual account (see Figure 3C).
  Further system control tasks, such as changing the language, password, or location, can also be handled via the smartphone app. The language settings can either be accessed via the landing page (see Figure 4A) or the general app settings (see Figure 4B). The general app settings are always accessible via a menu icon on the top left (see Figure 4C).
  The core function of the app is a control center through which various training interventions (e.g., cognitive training) and chatbots can be started. For this, the dashboard functions as a single point of contact (see Figure 5A). From there, other components of the e-VITA architecture can be accessed (see Figure 5B). The dashboard will provide links to, for instance, data analytics, profile data, performance indicators, status of the different interactive devices, privacy, and security.
  Users may want to use chatbots, social platforms, privacy dashboards, or other applications outside their home. To enable this, components of e-VITA that can be reached via URL can be accessed from outside the home, given that an internet connection is available. The dashboard consists of a grid layout with labeled tiles that link to other components of the e-VITA architecture. It is available in all languages of the e-VITA project. Users will observe this page after logging into their account. It offers a quick overview of all contents that can be accessed from the app.
  Chatbots are software agents that use a dialog function, such as a text or speech interface, and are based on natural language processing (NLP). The chatbot can be used to extract information from a user’s statement or input. The chatbot is also connected to the e-VITA dashboard. The chatbot in itself is a Telegram account that can automatically answer the text messages sent to it. To start a conversation with these chatbots, the user can either type ‘/start’ or press the start button on the screen. Once a piece of text is sent by the user to the chatbot, the message, the system starts to search for the intent and extracts entities from the user’s sentence. At the same time, the previous messages are also taken into consideration for extracting information that the user might have asked for before. Information is then returned to the middleware, where the request is further processed.
- *Social Platform*: The social platform allows the users to register and enroll in groups. Groups are meant to be interest areas. Users may either contact or create groups, based on their personal interests (e.g., nord walking and cooking classes), and may also get located to set up feeds, based on the country. Furthermore, users may be notified of upcoming meetings from groups and agenda updates of meetings. The main purpose is to establish a bridge between youth and older adults and between individuals and communities by creating a social platform in which different types of activities (e.g., cultural, sport, cooking, repairing, sewing, and gardening) are carried out to stimulate the users in remaining active. To make it efficient and easy to get connected, living labs, coaches, and those who offer their services will operate based on their location, which will be available only for the local community. Social platform application is maintained based on the countries and different communities (more specifically the local location and helping community directly around the primary end-users), to offer user services in the interest of the area. Living laboratories and international study site locations will be available to offer suitable activities to the users based on their location. For this purpose, in HumHub, Google location services will be used. Social platform operates as a website that can be accessed easily on any smartphone, tablet, and computer.
- Sign up: The application will ask to get permission of location to detect the user’s country to set up feeds based on the country. The user will enter credentials on the homepage, once registered.
- Registration: Upon registration, the user has designated roles such as administrator, service provider, or community organization (secondary stakeholders). Administrators are users who have technical responsibilities for the maintenance of the website, while service providers are users who offer either voluntary help or coaching. Community organizers are users who arrange local activities, such as meetups.
- Groups: There are different interest areas based on groups. Users may contact these groups or create groups based on their interests by themselves.
- Notification: In this section, the system will notify upcoming meetings from groups and agenda updates of meetings (update about the new updates of the app also). Notifications can be turned off in Settings.
- Messages: Users may contact people who initiate the activities or get contacted by the manager of those activities by getting detailed information through the chat system. When the users receive a message, they will be notified by message, such as email, and it will be marked on the top of the page. When a user clicks on the “Message” button, he/she will get a short description of the message, and in order to reply, users have to go to the “Conversation.”
- Feed: On feeds, users observe a variety of group meetings in the upcoming week. Moreover, they can review that volunteer announcements offer different services. The feed page is also considered as the main page, and users observe a review of the last activities.
- Settings: Users will be able to change location, language, and password and be able to log out. Furthermore, it is possible for the user to change or delete profiles on this page. Users can offer different types of services and, at the same time, benefit from offered services. Types of services will be offered under different categories. Users can also change their personal information on this page. This information includes profile photo, Email, and location. To make different changes to the profile directly, users have to click on their username, which is on the top of the page. The pop-up list will be shown, in which users choose “Settings” to make desired changes. On these settings, user can choose their desired language to use the application. To change the language, users have to choose “Settings,” and a core settings list will be shown, in which the “Language option” will be listed.

**Figure 6:**
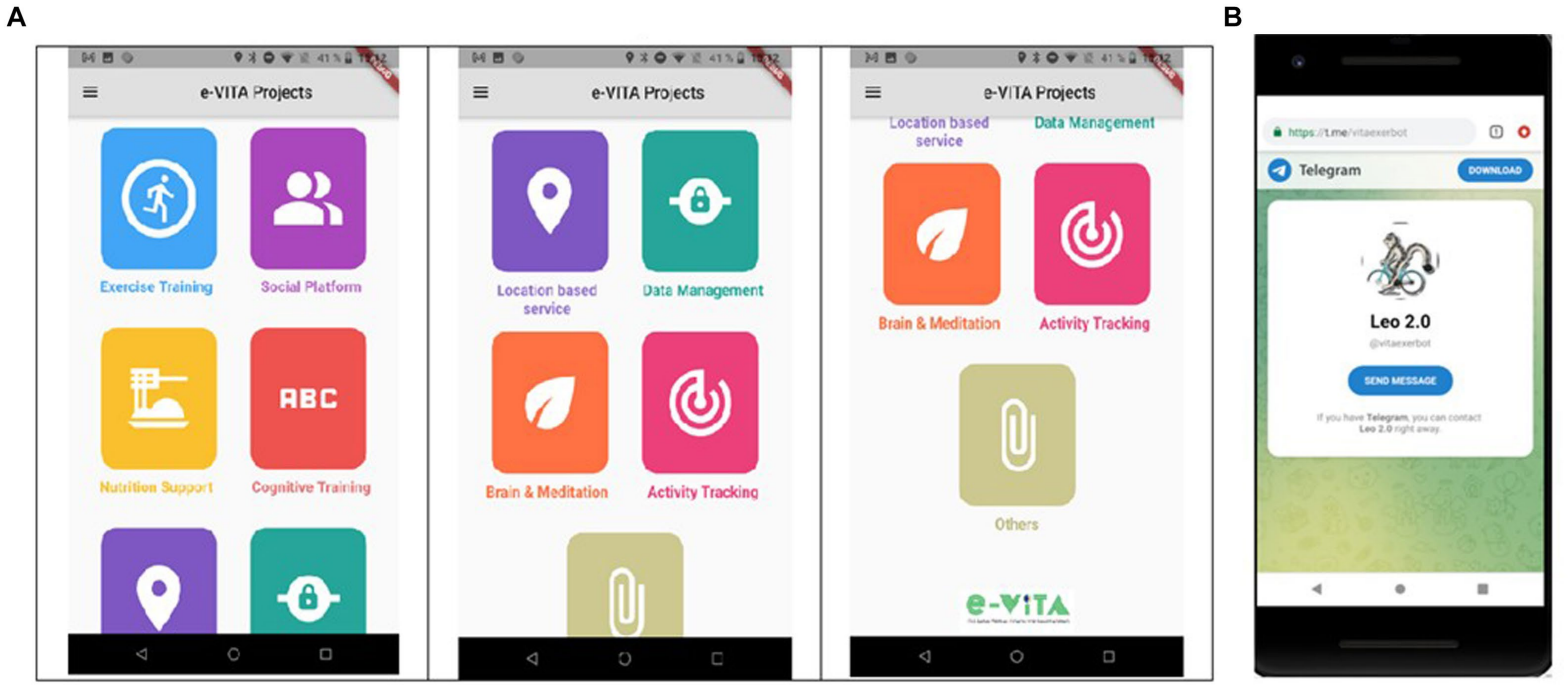
Screenshots of social platform—**(A)** thematic groups of interests to which the user can subscribe and **(B)** view of the members of one group.

Figure 6 shows some screenshots of the Social Platform.

### Use case configurator (UCC)

The Use Cases Configurator (UCC) stands as an independent software component within the e-VITA platform, which is tasked with translating user needs, environmental requirements, and configuration settings into technical specifications for the sensing and coaching system. This configurator prioritizes the creation of a smart living environment that balances cost-effectiveness and sensor efficiency, ensuring accurate measurements while identifying optimal devices for virtual coaching. The overarching goal is to deliver a service tailored to users’ needs and preferences. The UCC features a graphical interface, offering insights of end-users into the composition of the e-VITA platform based on their selected information, preferences, and goals. Inputs for the configurator include the needs and requirements of older adults, encompassing details about the living environment (house architecture, rooms, etc.), living situation (single or multiple residents), sensor acceptance (wearable or stationary sensors), privacy settings, and personal information (gender, age, and religion).

### Emotion detection system (EDS) module

The e-VITA Emotion Detection System (EDS) module will serve as one of the basic foundations for an empathic coaching system. That is, by accurately detecting the end-users’ emotional state, the EDS enables subsequent, high-level components to flexibly adjust their functioning and end-user interaction, in order to achieve a higher level of acceptance, usability, and well-being. The EDS layer imports audio samples from the current speech of the user (pseudonymized) during the interaction with the coaching devices via speech. Then, it pre-processes these data and decomposes the audio signal into its statistical sub-components (anonymized, no reverse-engineering possible). Based on this, a classification layer detects the currently most prominent emotion from a fixed set of basic emotions. Another approach will be to add a sentiment analysis to the EDS. This means, that in addition to the acoustic features of the audio data coming from the participant, information is extracted that is related to the participant’s sentiment or opinion, based on Natural Language Processing (NLP). For this, the audio data will be transcribed to a text, which will be then analyzed based on keywords, that represent a basic emotion. This information will be then added to the results of the acoustic analysis, leading to a final model with labels of basic emotions such as anger, disgust, fear, joy, neutral, and sadness. As a result, components, directly modeling the interaction with the end-user, for instance, dialog management, can then be augmented to fit the user’s emotional state.

### Outcome measures

All outcomes will be measured following a standardized operating procedure. The primary endpoint of the study is constituted by the improvement in the quality of life after the use of the e-VITA system, to be measured through the EQ-5D-5L, after 6 months of training.

The secondary endpoints encompass: (1) adherence to the coaching intervention, measured by the frequency of technological device usage, and collected through semi-structured interviews and technical data; (2) usability and the user experience of the overall system assessed using the System Usability Scale (SUS) (62) at both the midpoint and the conclusion of the experiments, along with the User Experience Questionnaire [UEQ (63) and UEQ+ (64)] in the middle and at the end of the experiments; (3) improvement in eHealth literacy, to be addressed through the eHealth Literacy Scale (eHEALS) (65), social connectedness through the UCLA Loneliness scale (UCLA) (66), nutritional state through the short version of Food Frequency Questionnaire (FFQ) (67) to estimate the frequency of daily food intake over a period, cognitive status, through Montreal Cognitive Assessment (MoCA) (39), psychological mood through the Geriatric Depression Scale (GDS) (40), functional status through Short Physical Performce Battery (SPPB) (41), participation to leisure activities through an *ad-hoc* checklist; meets the participant’s objectives through ADTPA-5 (scale B and E adapted) (68). A semi-structured interview will be used to complement the information collected by questionnaires, focusing on acceptability, attitude, usability, and cost–benefit analysis; other questionnaires will be used to better understand our participants’ sociodemographics questionnaire, Clinical Frailty Scale (CFS) (38), Big Five Inventory – 10 (BFI-10) (69), and Affinity for Technology Interaction (ATI) (70).

Table 3 presents a concise overview of all collected data and their respective collection time points.

**Table 3:** Schedule of assessment and outcome measures.

Dimension	Scale	R	T0	T1	T2
Clinical frailty scale	CFS	X			
Cognitive status	MoCA	X			X
Psychological status	GDS	X			X
Physical capacity	SPPB	X			X
Overall health status	Clinical anamnesis	X			
General information	Socio-demographics questionnaire	X			
Quality of life	EQ-5D-5L		X		X
Goals and expectation	ATDPA-5 (Scale B and E)		X		X (Scale B)
eHealth literacy	eHEALS		X		X
Social connectedness	UCLA		X	X	X
Personality	Big Five Inventory – 10 items		X		
Nutrition	Short FFQ		X		X
Adherence	Collected through the system and interview			X	X
Leisure activities	Physical and leisure activity checklist		X	X	X
Usability	SUS			X	X
User experience	UEQ, UEQ+			X	X
Attitude, usability, acceptability and cost–benefit analysis	Semi-structured interview				X
Affinity for technology interaction	ATI		X		

R, recruitment; T0, first evaluation; T1, intermediate evaluation; T2, final evaluation; CFS, Clinical Frailty Scale; MoCA, Montreal Cognitive Assessment; GDS, Geriatric Depression Scale; SPPB, Short Physical Performance Battery; ATDPA, Assistive Technology Device Predisposition Assessment; FFQ, Food frequency questionnaires; SUS, System Usability Scale; UEQ, User Experience Questionnaire; ATI, Affinity for Technology Interaction.

The scales which will be used during the evaluations are described below.

#### Clinical frailty scale (CFS)

This descriptive scale divides the older participants into nine classes based on the information provided by them and their relatives: between 1 and 3, the patient is non-frail, pre-frail if 4, and he is frail from 5 to 9.

#### Montreal cognitive assessment (MoCA)

Montreal Cognitive Assessment (MoCA) serves as a validated cognitive test, which is recognized for its high sensitivity in the early detection of mild cognitive impairment (MCI). MoCA efficiently assesses various cognitive domains, including short-term memory, visuospatial abilities, executive functions, attention, concentration, working memory, language, and orientation to time and place. The final version of MoCA, accessible at www.mocatest.org, is a 1-page, 30-point test that can be administered in just 10 min. Specific details regarding the MoCA items are as follows: (1) Short-Term Memory Recall Task (5 points): involves two learning trials of five nouns and delayed recall after approximately 5 min; (2) Visuospatial Abilities (4 points): clock-drawing task (3 points), three-dimensional cube copy (1 point); (3) Executive Functions (4 points): alternation task adapted from the Trail Making B task (1 point), phonemic fluency task (1 point), two-item verbal abstraction task (2 points); (4) Attention, Concentration, and Working Memory (6 points): sustained attention task (target detection using tapping; 1 point), serial subtraction task (3 points), digits forward and backward (1 point each); (5) Language (8 points): three-item confrontation naming task with low-familiarity animals (lion, camel, and rhinoceros; 3 points), repetition of two syntactically complex sentences (2 points), phonemic fluency task; (6) Orientation to Time and Place (6 points): assessment of orientation across various dimensions. The comprehensive nature of MoCA allows for a thorough examination of cognitive abilities in a relatively short time frame.

#### Geriatric depression scale (GDS) 5-item version

This questionnaire assesses the current condition of the patient’s mood. For the screening required by our study, only the first five items of the scale can be used. The answers highlighted indicate the statements expected by a non-depressed subject.

#### Short physical performance battery (SPPB)

It assesses physical performance on the basis of three criteria by testing balance, walking speed, and chair-raising abilities. This scale is used for the inclusion of participants (40).

#### EQ-5D-5L

The scale consists of five dimensions: mobility, independence, usual activities, pain/discomfort, and anxiety/depression. Each dimension has five levels: no problems, mild problems, moderate problems, severe problems, and extreme problems. The participant is asked to indicate his/her health status by ticking the box corresponding to the most appropriate statement in each of the five dimensions. The numbers from the five dimensions can be combined into a 5-digit number that describes the health status of the participant.

#### Assistive technology device predisposition assessment (ATDPA-5 – scales B and E)

This scale assesses the person’s need for technology. It has two parts. A part on the individual with 9 items assessing functional capacities and 11 items on well-being. These first 20 items are to be filled in on a five-point Likert scale, ranging from 1: poor/not satisfied to 5: excellent/very satisfied. Finally, this last part also assesses personal and psychosocial characteristics. There is no threshold value for these last items. The second part deals with technological tools with 12 items, highlighting their expectations in terms of benefits toward three technological tools. There is no threshold for this scale, but the scores range from 0 to 60 (sum of the statements). The tool with the highest score is considered the most important. This scale is used at the beginning of the experiment. Only parts B and E will be used and adapted for the project (68).

#### Big five inventory – 10 (BFI-10)

The BFI-10 (69) is a concise 10-item scale designed to assess the Big Five personality traits: extraversion, agreeableness, conscientiousness, emotional stability, and openness. Participants rate each item on a scale ranging from 1 (disagree strongly) to 5 (agree strongly).

#### Short food frequency questionnaire (FFQ)

FFQ serves as a scale to estimate the frequency of daily food intake over a specified period. This questionnaire seeks information on how often certain foods are consumed (e.g., once daily, once or twice a week, and once or twice a month) and the approximate serving size. It is designed to capture data on habitual food intake rather than quantifying the exact nutrient amount ingested. In Europe, partners will utilize the scale developed by Robinson et al. (67).

#### eHealth scale

The eHEALS is an 8-item assessment designed to gauge eHealth literacy, measuring consumers’ collective knowledge, comfort, and perceived skills in finding, evaluating, and applying electronic health information to address health-related issues.

#### Revised UCLA loneliness scale version 3

The revised UCLA Loneliness Scale Version 3 is a dependable and consistent questionnaire-based measure designed to assess trait loneliness (66). Comprising 20 items, respondents rate each item on a scale from 1 (never) to 4 (often), yielding a loneliness score ranging from 20 to 80. A higher score on the scale indicates a greater level of trait loneliness.

#### System usability scale (SUS)

The System Usability Scale (SUS) stands as a reliable tool for assessing usability, featuring a 10-item questionnaire with five response options ranging from ‘strongly agree’ to ‘strongly disagree’. This versatile scale enables the evaluation of a diverse range of products and services, spanning hardware, software, mobile devices, websites, and applications. Its ease of administration to participants, suitability for small sample sizes, and ability to effectively distinguish between usable and unusable systems contribute to its widespread applicability and reliability in usability assessments.

#### User experience questionnaire (UEQ)

The questionnaire incorporates scales that provide a comprehensive assessment of the user experience. It encompasses classical usability dimensions such as efficiency, perspicuity, and dependability while also evaluating user experience aspects, such as originality and stimulation. This holistic approach ensures a well-rounded understanding of the overall user experience, considering both functional and experiential aspects of interaction with the system or product.

#### User experience questionnaire + (UEQ+)

To measure the user experience of the voice interaction in particular, additional scales such as response behavior or response quality from the UEQ+ can be added.

#### Affinity for technology interaction (ATI)

The scale, evaluated in 2019 from the study by Franke et al., measures a person’s interaction-related affinity with technology. It consists of nine items and uses a six-point Likert scale ranging from 1 = completely disagree to 6 = completely agree (71).

#### Physical and leisure activity checklist

An *ad-hoc* checklist has been developed to collect information on engagement in leisure and physical activities of the participants during the e-VITA trial. It contains questions, which are rated on a six-point Likert scale, about common physical and leisure activities and a section to report the personal practices and frequency put in place by the participants.

#### Scales at T0 and T2 for the control group

These two scales are necessary to better understand our participants. The scale at T0 focuses on the dimensions of daily life, social, and prevention. The T2 scale focuses on the participants’ feelings, following the experiment, their experience with the information booklet and its usability, and their well-being.

### Risk–benefit analysis

During the use of robotic platforms or, in wider terms of technological applications, a general difficulty in distinguishing between the artificial world and reality may occur. Especially, in the case of vulnerable older adults, the interaction may generate a general feeling of attachment and dependency. To avoid this, e-VITA has been designed to not look like a human but to preserve artificial aesthetics, as suggested by the guidelines. In addition, during the first contact with the participants but also during all the interventions, researchers will be in contact with the users, continuously stimulating awareness about the technological applications and monitoring the appropriateness of the use of the solution in terms of autonomy of the users, specific needs, and personal preferences. In particular, following the recommendations provided by Comitato nazionale di bioetica (Cnb) of the Ministry of Health (71) on robotics and roboethics, the exit strategy of e-VITA will aim to:

- promote adequate experimentation of robotics in the field assistance in order to ensure conditions for the physical and psychological integrity of the user, explaining the risks and benefits, which was highlighted also in the informed consent;
- ensure both equitable access to robotic and general technologies and the use of robots to assist and not to replace humans, in order to avoid delegating the irreplaceable human task of care and assistance to the machine;
- the need that the introduction of robotics in medicine entails always the real consideration of the benefits, of the complexity of the change complete with the structure of the services and the economic burden that this entails.

There is a risk that the older adults may wish to stop interacting with the technological devices (for example, because they do not like the NAO robot). In this case, the experiment will be immediately stopped and terminated.

The presence and use of technological devices (virtual coaches and sensors) in participants’ homes can be a source of discomfort. Therefore, pre-studies with end-users (older adults) and stakeholders (informal carers, health professionals, family, NGOs etc.) have been carried out in order to propose virtual coaches that meet the needs of older adults. In addition, safety procedures were also designed to limit the risks as much as possible.

To protect the safety of participants:

- participants will be informed about the appropriate use of the virtual coach or technological device (e.g., cannot lean on the technological device or make a movement that could destabilize it);
- the researcher will train the participant in the use of the sensors and be available in case of problems;
- the technological devices will be placed in the participant’s home in a configuration that allows them to be used safely.

In the case of adverse events occurring despite the precautions described above:

- participants will be instructed to press the “off” button on the device or to disconnect it, according to the instructions in the user manual;
- participants will call the researcher, who will assist the participant in case of problems;
- participants will be able to call the researcher, who will come and ensure that no damage has been caused to the participants or to any other person.

To mitigate potential technological dependency, especially with humanoid or theomorphic robotic systems, participants will undergo instruction and training for the appropriate and limited use of virtual coaches. Researchers will maintain contact with participants to ensure proper guidance. Despite the existing risk, it is crucial to acknowledge that current technology has not reached the level of sophistication, which is necessary for natural human–robot interaction. Presently, there is limited progress in developing coaching devices that are capable of minimal social interaction, involving emotional and psychological engagement in controlled conditions. However, precautions, including the provision of adequate training and daily support/monitoring by researchers, are essential to safely use coaching devices in emotional, social, and psychological terms and prevent potential future dependency.

Finally, if after the experimentation, the participants would ask for a longer use of the system, they will be asked to be involved in similar studies, to ensure the continued use of the technology. Moreover, after the end of the study, the opportunity to receive personalized support on everyday technology will be offered to the participants about eHealth literacy and similar solutions for health.

### Data management

The project is dedicated to upholding the anonymity and confidentiality of participants throughout all stages, encompassing screening, recruitment, testing, evaluation, and dissemination procedures. Data collection, usage, and storage strictly adhere to national laws, the General Data Protection Regulation (GDPR) of EU, and APPI. Participants retain rights, such as access, information, withdrawal, and data erasure. Additionally, servers are situated in the European Union and comply with the GDPR standards. Data collection follows the principle of data minimization, ensuring that personal information collected is directly relevant and necessary for the specific goals of testing and evaluation. Specific software with blocks and data entry checks is employed to minimize entry errors. All screening data are discarded upon project completion. During testing procedures, any visual, auditory, and sensory data processed by the robot are discarded after completing the procedures, except for the number of interactions logged with each participant, which remains anonymous. After the conclusion of this project, all research data will be openly available for secondary analysis after 3 years.

### Data analysis

A comprehensive analysis plan will be established to serve as the foundation for conducting subsequent analyses. This plan will outline the methodologies, procedures, and criteria for conducting the analyses, ensuring a systematic and well-organized approach to data interpretation and result generation.

#### Data collected by the researchers

The first step of the data analysis will deal with the description of the sample. Continuous variables will be reported as either mean and standard deviation or median and interquartile range based on their distribution (assessed using the Kolmogorov–Smirnov test). Categorical variables will be expressed as an absolute number and percentage. Comparison of baseline measurements between groups will be evaluated by unpaired t-test (for normal distribution), Mann–Whitney U tests (for non-normal distribution), or chi-square tests (for categorical variables). Within each group, independent and dependent variables will be compared between the pre-conditions and post-conditions using the same tests as appropriate. The treatment effect on the outcome variables will be evaluated by using repeated measures ANOVA, in order to compare the changes over time in the outcome measures between the intervention group and control group. Moreover, a linear regression model on the outcome variation between baseline and follow-up will be estimated in order to evaluate the effect of the treatment adjusted for all potential confounders. For the analysis of the results, the two types of subjects, stratified according to the MoCA cutoff (score ≥ 26 for cognitively healthy subjects, score 22 to 25 for subjects with mild cognitive decline), will be considered separately. Descriptive statistical analyses will be performed on the quantitative data with SPSS or R studio.

#### Data collected by the technological devices

One of the uses of the data collected from the sensors is to infer the activity of the user and know his or her location so that the voice coach carries out personalized dialogs adapted to the contexts of the user. The environmental data will be used to identify dangerous situations and difficulties and inform the user by giving him the right recommendation to remedy this situation. The aggregation of activity data from several testing centers will serve to motivate users and strengthen their adherence to the experiment. The analysis of user activity data and their interactions at well-defined milestones in the experiment will make it possible to detect system failures as early as possible, in order to prevent the user from dropping out.

### Ethical consideration

The study received approval from the Ethics Committee of the Istituto Nazionale Ricovero e Cura per Anziani (IRCCS INRCA) (CE INRCA 23005), the German Association for Nursing Science (Deutsche Gesellschaft für Pflegewissenschaft e.V.) for the Diocesan Caritas Association of the Archdiocese of Cologne, and the Research Ethics Committee (Comité Ethique de la Recherche) for Assistance Publique – Hôpitaux de Paris. It was registered in ClinicalTrials.gov on 28 April 2023, with the number NCT05835856.

The study will be adhered to regulatory and legal requirements, which were initiated after receiving evaluation and approval from an independent ethics committee and completing administrative requirements at the conducting institution.

Additionally, all potentially eligible participants will receive comprehensive information about the study and must provide consent to participate.

Participants are required to consent to the processing of personal data in anonymous and aggregate form, aligning with EU Regulation 2016/679 (GDPR)/APPI on the protection of individuals and concerning the processing of personal data and Legislative Decree No. 101/2018. The participant must be informed that his or her data may be examined by authorized personnel or members of the competent ethics committee and officials of the competent regulatory authorities;

The participant is also informed and asked to provide *ad-hoc* informed consent to participate in the study, including data retention for up to 10 or 15 years (depending on the country’s rules) after completion of the study.

Each signature must be personally dated by each signatory, and the informed consent and any additional patient information must be retained by the investigator. A signed copy of the informed consent and information sheet will be given to each patient.

Participant information and consent forms are included in the documentation attached to the request for approval by the local ethical committee.

[Optional] The Participant has the opportunity to indicate his or her agreement to the retention and use of his or her data long after the end of the project under the OPEN ACCESS TO SCIENTIFIC PUBLICATIONS AND OPEN RESEARCH DATA as requested by the European Commission.

In the documentation submitted for approval by the IRCCS INRCA Ethics Committee, patient information and consent forms are integral components. These documents play a crucial role in ensuring that participants receive detailed information about the study and have the opportunity to provide informed consent before their involvement. This approach aligns with ethical standards and regulatory requirements to safeguard participants’ rights and well-being.

### Dissemination of research findings

The study findings will be utilized for publication in peer-reviewed scientific journals, contributing to the academic and research community’s knowledge. Additionally, the results will be presented in scientific meetings, fostering discussions and knowledge sharing within the academic and professional spheres. Summaries of the outcomes will be provided to investigators, enabling them to disseminate key findings within their clinics, further promoting the practical application of the study results in relevant healthcare settings.

## Discussion

In the face of an aging population, interventions are needed to reduce or minimize the consequences of various diseases related to advancing age (6), support the healthcare system, and promote active aging, in which the senior is allowed to maintain their autonomy as long as possible. One such solution has been identified in virtual coaches (16) which, through a personalized system that constantly keeps the senior constantly monitored (23) identify situations in which it would be desirable to intervene and propose to the user different solutions and activities adapted to his or her needs and requirements, allowing the person to maintain a healthy and active lifestyle. Despite the advantages that these technologies bring, they also have some limitations: in fact, there are several coaches of this type, but it is obviously necessary for them to be perfectly integrated into the lives of the older adults so that they take into account their real needs that are also due to belonging to different cultures. It is from this premise that the e-VITA project begins, in which different technological devices were proposed to consider people’s actual needs; the present study adopts a randomized, single-blind controlled trial design to enlist healthy older adults aged 65 years and older. Its primary objective is to assess the effectiveness of the e-VITA platform in this demographic. This robust research design allows for rigorous examination and comparison of outcomes, ensuring a comprehensive evaluation of the platform’s impact on the health and well-being of the participants. Participants will be selected from the four sites described above. In this study, the primary goal is to assess the improvement in the quality of life (QoL) of older individuals, which was measured after 6 months. To evaluate the effectiveness of the treatment, the study population will be stratified into two groups. The experimental group will engage with the e-VITA platform, comprising a virtual coach, various monitoring sensors, a smartphone for home use, and a booklet. In contrast, the control group will receive the booklet alone. This design enables a comparative analysis, allowing for the examination of the impact of the e-VITA platform on enhancing the quality of life in older adults. The virtual coaching system will detect daily physical, cognitive, psychological, and social changes in the older person and provide useful advice and recommendations accordingly. In this way, although the primary goal is to improve the person’s quality of life, the system will also act on other dimensions, such as mood, cognitive status, nutrition, social connectedness, and eHealth literacy. In this study, the intervention will be regularly monitored by the research team, which will conduct evaluations in order to ensure its quality. In addition, researchers will be constantly over the call for any doubts or difficulties from users to support their motivation and participation in the trial.

### Limitations

Participants will be selected from the Neurology Operating Unit and the Rehabilitation Medicine Operating Unit of IRCCS INRCA, so the results may not be generalizable to the general population. In addition, the e-VITA system is composed of several types of technological equipment, which could be a limitation for those older adults who, while meeting the inclusion criteria, are not particularly familiar with these types of devices. These problems could be solved by incorporating user-friendly interfaces with clear and intuitive navigation to enhance ease of use. In addition, comprehensive guides and tutorials could be developed to help participants familiarizing themselves with the system. Moreover, users could attend training sessions to be guided through initial set-up and use, to accommodate those who may need further assistance.

## Conclusion

The study aims to introduce users to a cross-cultural and customizable virtual coach, which was designed to address various aspects of the older person’s well-being, including social relationships, physical activity, autonomy, and safety. The ultimate goal is to enhance the overall quality of life for older individuals. To rigorously assess the true potential and identify any potential issues with the intervention, a randomized controlled trial is deemed essential. This research design will provide valuable insights into the effectiveness and challenges associated with the implementation of the virtual coach in improving the well-being of older adults.

## Author contributions

RoB: Conceptualization, Methodology, Writing – original draft, Writing – review & editing. VS: Conceptualization, Writing – review & editing. GA: Data curation, Formal analysis, Writing – review & editing. AM: Data curation, Writing – review & editing. MB: Data curation, Writing – review & editing. FB: Data curation, Formal analysis, Writing – review & editing. EM: Methodology, Writing – original draft, Writing – review & editing. A-SR: Conceptualization, Writing – review & editing. SD: Writing – review & editing. CP: Conceptualization, Writing – review & editing. JM: Conceptualization, Writing – review & editing. RyB: Conceptualization, Writing – review & editing. TO: Conceptualization, Writing – review & editing. RW: Conceptualization, Supervision, Writing – review & editing.
